# The Prognostic Role of Red Blood Cell Distribution Width in Coronary Artery Disease: A Review of the Pathophysiology

**DOI:** 10.1155/2015/824624

**Published:** 2015-08-26

**Authors:** Kamil Bujak, Jarosław Wasilewski, Tadeusz Osadnik, Sandra Jonczyk, Aleksandra Kołodziejska, Marek Gierlotka, Mariusz Gąsior

**Affiliations:** 3rd Department of Cardiology, SMDZ in Zabrze, Medical University of Silesia, Marii Skłodowskiej Curie Street 9, 41-800 Zabrze, Poland

## Abstract

Red blood cell distribution width (RDW) is a measure of red blood cell volume variations (anisocytosis) and is reported as part of a standard complete blood count. In recent years, numerous studies have noted the importance of RDW as a predictor of poor clinical outcomes in the settings of various diseases, including coronary artery disease (CAD). In this paper, we discuss the prognostic value of RDW in CAD and describe the pathophysiological connection between RDW and acute coronary syndrome. In our opinion, the negative prognostic effects of elevated RDW levels may be attributed to the adverse effects of independent risk factors such as inflammation, oxidative stress, and vitamin D_3_ and iron deficiency on bone marrow function (erythropoiesis). Elevated RDW values may reflect the intensity of these phenomena and their unfavorable impacts on bone marrow erythropoiesis. Furthermore, decreased red blood cell deformability among patients with higher RDW values impairs blood flow through the microcirculation, resulting in the diminution of oxygen supply at the tissue level, particularly among patients suffering from myocardial infarction treated with urgent revascularization.

## 1. Introduction

Red blood cell distribution width (RDW) is a parameter routinely measured by most modern hematology analyzers. RDW is defined as the quotient of standard deviation of red blood cell volume and its mean volume and is expressed as a percentage according to the following formula: RDW = (standard deviation of red blood cell volume/mean cell volume) × 100. Higher RDW values reflect greater variations in red blood cell volume [1].

One of the first studies to assess the role of RDW in cardiovascular disease was published by Felker et al. in 2007. The authors noted the usefulness of RDW as a prognostic marker among patients with heart failure (HF) [2]. Subsequent studies have confirmed the significance of RDW as a predictor of mortality both in the general population [3] and in patients with various diseases, including peripheral artery disease (PAD) [4], chronic obstructive pulmonary disease (COPD) [5], and kidney failure [6–8]. In the literature, there exist increasing amounts of data describing the link between RDW and prognosis in patients with stable coronary artery disease (SCAD) [9], including patients undergoing percutaneous coronary intervention (PCI) [10] and patients suffering from myocardial infarction (MI) [11, 12].

The aim of this review is to describe the prognostic utility of RDW in patients with coronary artery disease (CAD) and to elucidate the mechanism underlying the relationship between elevated RDW values and poor patient prognosis in this particular group of patients.

## 2. The Prognostic and Diagnostic Value of RDW in CAD

In recent years, numerous papers have been published regarding the value of RDW in the risk stratification of patients with CAD [4, 9, 10, 12]. We have, therefore, searched PubMed and Scopus for English-language articles regarding the prognostic and diagnostic role of RDW in patients with CAD using the following keywords: red cell distribution width; RDW; coronary artery disease; myocardial infarction; acute coronary syndrome. Additionally, we have searched reference lists of publications in this field. The most important and appropriate studies investigating the prognostic and diagnostic value of RDW in patients with CAD are summarized in Table 1.

One of the studies that assessed the prognostic role of RDW was conducted at our center. We demonstrated that RDW was an independent risk factor for long-term mortality in patients undergoing PCI for SCAD [10], and CAD patients with elevated RDW values more frequently suffered from concomitant diseases such as PAD and COPD [10]. Furthermore, RDW itself correlates with both the severity and the complexity of coronary artery atherosclerosis, as determined using the SYNTAX [43] and Gensini scores [40].

Tonelli et al. determined that RDW was a risk factor for MI, stroke, and symptomatic HF in patients with CAD [9]. Elevated RDW levels are also associated with increased in-hospital and long-term mortality among patients suffering from MI [11, 12]. Lippi et al. observed that RDW had diagnostic value in patients admitted to the intensive care unit for chest pain suggestive of acute coronary syndrome. Combined measurements of both troponin T and RDW have allowed for diagnosing MI with greater sensitivity than the analysis of troponin T alone [36]. In patients with non-ST elevation myocardial infarction, RDW may be used to predict the occurrence of fragmented QRS complexes noted on electrocardiography [46] and the absence of collateral circulation on coronary angiography [47]. Additionally, there exist publications that describe the relationship between RDW and the lack of tissue reperfusion in patients with MI treated via PCI [44, 57].

The results of large, prospective studies with long-term follow-up periods (Tromsø and National Health and Nutrition Examination Survey) show that elevated RDW values increase the risk of MI and mortality due to CAD in the general population, regardless of other known CAD risk factors [67, 69], including the risks of MI and mortality among elderly patients without aging-associated diseases [3].

One of the most important limitations affecting the long-term outcomes of CAD invasive treatment is restenosis [70] which occurs more frequently in patients with elevated RDW values, which is the case following both bare-metal [61] and antimitotic drug-eluting stent implantation [64]. RDW is also valuable in assessing the risk of major bleeding complications in patients undergoing PCI [60], as well as the risk of contrast-induced nephropathy [65]. In patients undergoing coronary artery by-pass grafting, RDW is a risk factor for atrial fibrillation following cardiac surgery [59].

One limitation to the clinical usefulness of RDW in predicting adverse clinical outcomes is its moderate sensitivity and specificity [12]. However, these parameters are often not worse than any other laboratory parameters used in risk stratification, including C-reactive protein (CRP) [67, 71]. Additionally, there are no unambiguous RDW cut-off values. In papers concerning this problem, the cut-off values vary depending on the population and adverse events studied [72]. The potential use of RDW as a predictive factor for cardiovascular events appears to reside in its utilization as an element of various risk scores.

The main limitation of the vast majority of cited studies is that they were observational and described only the statistical relationship between elevated RDW value and outcomes of patients with CAD, but not the pathophysiological mechanism explaining that phenomenon. Moreover, only a few published studies included factors influencing the hematopoiesis, that is, levels of folic acid, vitamin B12, markers of inflammation, and iron status as potential confounding factors [72, 73].

In summary, RDW is a significant predictor of both all-cause mortality and adverse cardiovascular events in patients with CAD. Increased values of this parameter are associated with greater numbers of comorbidities and a higher likelihood of complications among patients with CAD treated via PCI.

In the following sections, the potential mechanisms explaining why elevated RDW levels are a strong, negative predictive factor among patients with CAD will be discussed.

## 3. The Reasons for the Poor Prognosis Observed in Patients with CAD with High RDW Levels

The influence of RDW on prognosis among patients with cardiovascular disease has been extensively studied [9–12]. Nonetheless, the reasons for the poor prognosis in this patient group remain unclear. For instance, it has not been determined whether RDW is only a marker of the severities of various disorders or if there is a direct pathophysiological relationship among anisocytosis, CAD, and poor prognosis in patients with CAD. Most likely, numerous factors impairing bone marrow hematopoietic function, which are identical to the factors that worsen the prognoses of patients with CAD, play integral roles in this process.

### 3.1. Anemia, Iron, and RDW

Anemia is a well-documented mortality risk factor among patients with CAD and is prevalent in cases of elevated RDW values [74, 75]. However, elevated RDW values increase mortality in patients with CAD regardless of their baseline hemoglobin levels [10, 16]. Furthermore, RDW has prognostic value in patients without anemia [10]. On the other hand, Salisbury et al. indicated that baseline RDW is associated with development of moderate-severe anemia during hospitalization in patients with MI and without concomitant anemia at admission [76]. It is not surprising that RDW is affected by iron metabolism [73]. Grammer et al. determined that reduced iron stores, regardless of hemoglobin concentrations, increased the likelihood of coronary artery atherosclerosis [77]. Furthermore, Ponikowska et al. determined that disturbances in iron metabolism increased mortality among patients with CAD and diabetes mellitus [78]. Perhaps iron status and low hemoglobin levels are only partially responsible for the poor prognoses observed among patients with elevated RDW levels.

### 3.2. Lipid Abnormalities and RDW

There are reports indicating that elevated RDW values correlate with unfavorable lipid profiles [79, 80]. Some insight into the relationship between RDW and lipid disorders was provided by Tziakas et al., who described a link between RDW and total cholesterol erythrocyte membrane (CEM) levels [81]. CEM level increases are responsible for the deterioration of cell deformability, which affects the lifespans of circulating erythrocytes, and this results in greater cellular turnover and elevated RDW values [82–84]. Previous studies have confirmed that once the necrotic core of the plaque accumulates erythrocytes, elevated CEM levels may result in plaque instability, which suggests that red blood cells may actively contribute to both plaque growth and plaque destabilization [85–87].

Lipid disorders decrease red cell membrane fluidity, and higher CEM levels result in the deterioration of blood flow through the microcirculation [88, 89]. This mechanism may explain the well-documented relationship between red blood cell rheology and the lack of tissue reperfusion following PCI in patients suffering from MI [56, 90]. This effect may also explain the slow flow phenomenon observed in the epicardial coronary arteries in symptomatic patients without coronary artery stenosis [91].

Previous studies have noted that statin therapy increases erythrocyte deformability [89, 92, 93]. However, Kucera et al. demonstrated that 12 weeks of atorvastatin therapy did not affect RDW levels despite improved lipid profiles [80]. It is possible that the statin therapy in question was not administered long enough to decrease erythrocyte membrane cholesterol concentrations (the mean erythrocyte lifespan is 115 days) [94].

### 3.3. Chronic Inflammation and RDW

Another explanation for the relationship between RDW and prognosis may be chronic inflammation. It is believed that even low-intensity inflammation plays a crucial role in atherogenesis and may be responsible for platelet activation [95, 96]. Lippi et al. described the relationship between RDW and inflammatory markers such as the erythrocyte sedimentation rate and high sensitivity CRP [97]. In previous studies, RDW correlated with interleukin-6, soluble tumor necrosis factors I and II receptor concentrations, and fibrinogen levels [73, 83, 98]. Additionally, chronic inflammation results in disorders of iron metabolism and decreases both the production of and bone marrow responsiveness to erythropoietin, resulting in impaired hematopoiesis and increased RDW levels [98–100]. The significance of both anemia and iron metabolism has been discussed previously.

### 3.4. Glycemic Disturbances and RDW

Veeranna et al. observed the correlation between HbA_1C_ levels (glycosylated hemoglobin) and RDW levels in patients without diabetes mellitus [101], and Lippi et al. observed the existence of a relationship between HbA_1C_ and RDW in an unselected population of elderly patients [102]. Additional studies have noted an increased prevalence of diabetes complications such as nephropathy and cardiovascular disease in patients with diabetes and elevated RDW levels [103, 104]. Garg et al. demonstrated that HbA_1C_ levels had an impact on CAD severity in patients without diabetes; therefore, these levels may be considered a mechanism linking RDW values to impaired glucose metabolism [105].

### 3.5. Vitamin D_3_ Deficiency and RDW

Another factor potentially affecting RDW is vitamin D_3_ deficiency, a well-established CAD risk factor [106]. Vitamin D_3_ deficiency is responsible for both cell proliferation and erythropoiesis, and vitamin D_3_ concentrations in the bone marrow are more than two hundred times greater than in the blood. Even a minor decrease in serum vitamin D_3_ levels may result in the derangement of bone marrow erythropoiesis [107, 108].

### 3.6. Oxidative Stress and RDW

Oxidative stress is responsible for shortening the lifespan of red blood cells, which intensifies both the production and the release of young cellular forms into the circulation, which is reflected by increased RDW levels [109]. Oxidative stress also generates oxidized low-density lipoprotein forms, which play an important role in atherogenesis [110]. Kobayashi et al. demonstrated that using a vitamin E-bonded hemodialyzer resulted in decreased RDW values in a group of patients with end-stage kidney disease undergoing hemodialysis. This effect was absent in patients dialyzed without vitamin E-bonded hemodialysis membranes [111]. The authors mentioned that the decreased RDW levels were accompanied by reduced whole-blood viscosity [111].

### 3.7. Renal Failure and RDW

Impaired kidney function increases mortality among patients with CAD [98, 112], and the relationship between low glomerular filtration rate, microalbuminuria, and elevated RDW levels has been demonstrated in previous studies [6, 8]. Impaired kidney function, however, is most likely not a separate mechanism linking elevated RDW values to poor patient prognosis in CAD [7], because, among patients with chronic kidney disease, chronic inflammation, greater oxidative stress, lipid disorders, vitamin D_3_ deficiency and anemia, and increased RDW levels are often noted [6, 8, 111, 113, 114].

### 3.8. Summary

The above-mentioned data indicate a correlation between RDW and other known atherosclerosis-predictive factors, although it is impossible to clearly identify the mechanism by which high erythrocyte anisocytosis serves as a negative prognostic marker in patients with CAD. In our opinion, the prognostic value of RDW results primarily from the negative impacts of inflammation and oxidative stress, as well as those of iron and vitamin D_3_ deficiency, on bone marrow erythropoiesis. Additionally, concurrent red blood cell deformability diminution may result in impaired flow through the microcirculation. It is also impossible to unambiguously ascertain which concomitant factors, including lipid, glycemic and iron metabolism disturbances, anemia, vitamin D_3_ deficiency, oxidative stress, inflammation, and the diminution of erythrocyte membrane deformability, are the primary causes of the poor prognoses observed in patients with CAD. In the authors' opinion, the predictive utility of RDW results from the summation of each of the above-mentioned factors' negative impacts on bone marrow erythropoietic function. Therefore, the prognostic value of RDW reflects the intensification of these phenomena.

## 4. Conclusions

RDW is an important risk factor for both mortality and cardiovascular events in patients with SCAD or acute coronary syndrome. It has not yet been determined whether anisocytosis is the cause of the poorer prognosis observed among these patients or if it is simply a marker of multiple pathological states connected with said prognosis. In contrast to the markers of inflammation and oxidative stress, which are not routinely analyzed, RDW provides valuable information concerning prognosis in patients with CAD.

## Figures and Tables

**Table 1:** Summary of the most relevant studies investigating prognostic and diagnostic value of RDW in patients with CAD.

Study	Type of study design	Study population	Main findings	References
RDW as a predictor of mortality and adverse cardiovascular events in patients with CAD				
Tonelli et al. (2008)	Post hoc analysis of data from a randomized trial	4,111 patients with CAD but no concomitant HF	RDW is an independent predictor of all-cause death, cardiovascular death, and cardiovascular events in patients with history of MI.	[9]
Poludasu et al. (2009)	Retrospective	859 patients who underwent PCI	Higher RDW level is an independent risk factor for death in patients undergoing PCI but only without anemia at baseline.	[13]
Nabais et al. (2009)	Retrospective	1,796 patients with ACS	Higher RDW level is an independent risk factor for 6-month death/MI in patients with ACS.	[14]
Dabbah et al. (2010)	Prospective	1,709 patients with MI	Baseline RDW and increase in RDW during hospitalization are associated with mortality in patients with MI.	[15]
Cavusoglu et al. (2010)	Prospective, cohort study	389 unselected, male patients who were referred to coronary angiography	RDW is a predictor of all-cause mortality in unselected patients referred to coronary angiography.	[16]
Wang et al. (2011)	Prospective	1,654 patients with ACS	RDW is a risk factor for death, heart failure, and recurrent MI in short-term follow-up.	[17]
Azab et al. (2011)	Retrospective	619 patients with NSTEMI	RDW is a predictor of long-term mortality in patients with NSTEMI.	[18]
Uyarel et al. (2011)	Retrospective	2,506 STEMI patients treated with primary PCI	RDW is a predictor of in-hospital and long-term cardiovascular mortality in patients with STEMI treated with primary PCI.	[19]
Lappé et al. (2011)	Post hoc analysis of prospective single-center registry	1,489 patients with angiographically documented CAD and 449 normal patients	RDW is a predictor of long-term all-cause mortality in both CAD and no-CAD patients.	[20]
Vaya et al. (2012)	Prospective	119 patients with MI	RDW is a predictor of recurrent cardiovascular events in patients with MI.	[21]
Gul et al. (2012)	Prospective	310 patients with NSTEMI or UA	RDW is independently associated with long-term cardiovascular mortality in NSTEMI/UA patients.	[22]
İlhan et al. (2012)	Retrospective	763 patients with MI treated with primary PCI	RDW is associated with in-hospital mortality but is not associated with impaired postinterventional TIMI flow.	[23]
Fatemi et al. (2013)	Post hoc analysis of prospective multicenter registry	1,689 patients who underwent PCI	RDW is an independent predictor of 1-year mortality and improves discriminative risk value in patients undergoing PCI.	[24]
Tsuboi et al. (2013)	Retrospective	560 diabetic patients with SCAD who underwent elective PCI	Increased RDW is associated with all-cause long-term mortality in diabetic patients undergoing elective PCI.	[25]
Warwick et al. (2013)	Post hoc observational analysis	8,615 patients who underwent isolated CABG	RDW is significantly associated with in-hospital and long-term mortality in patients undergoing isolated CABG.	[26]
Lee et al. (2013)	Post hoc analysis of prospective multicenter registry	1,596 patients with MI	Adding RDW to traditional risk factors significantly improves prediction for 12-month MACEs in patients with MI.	[27]
Ren et al. (2013)	Post hoc observational analysis	1,442 patients with SCAD	RDW is a predictor of 1-year mortality and 1-year ACS in Chinese patients with SCAD.	[28]
Osadnik et al. (2013)	Retrospective	2,550 patients with SCAD who underwent elective PCI	RDW is associated with comorbidity burdens and with long-term all-cause mortality in patients with SCAD undergoing elective PCI.	[10]
Yao et al. (2014)	Post hoc observational analysis	2,169 patients with CAD who underwent PCI with DES implantation	Elevated RDW is an independent predictor of mortality and cardiovascular adverse events in patients who underwent PCI with DES implantation.	[29]
Vieira et al. (2014)	Prospective	682 patients with ACS	RDW among other markers adds prognostic value to the GRACE risk score in patients with ACS and high risk defined by GRACE.	[30]
Sangoi et al. (2014)	Post hoc observational analysis	109 patients with MI	RDW has additional prognostic value on the GRACE risk score in prediction of in-hospital mortality.	[11]
Sun et al. (2014)	Retrospective	691 MI patients without HF at baseline	RDW predicts all-cause and cardiovascular mortality in patients with MI who are free of HF.	[12]
Arbel et al. (2014)	Post hoc analysis of prospective single-center registry	3,222 patients who underwent coronary angiography	RDW is independently associated with 3-year MACEs in consecutive patients referred to coronary angiography.	[31]
Arbel et al. (2014)	Post hoc analysis of prospective single-center registry	535 patients with STEMI treated with primary PCI	RDW above 14 is an independent predictor of long-term all-cause mortality in patients with STEMI undergoing primary PCI.	[32]
Bekler et al. (2015)	Retrospective	202 patients with NSTEMI or UA	Increased RDW is independently associated with long-term mortality in patients with non-ST elevation ACS.	[33]
Liu et al. (2015)	Retrospective	1891 patients ≥65 years old who underwent elective PCI	RDW is a predictor of intermediate-term all-cause mortality in elderly CAD patients treated with elective PCI.	[34]
Timoteo et al. (2015)	Prospective	787 patients with ACS	Inclusion of RDW in a model with GRACE risk score improves predictive value for all-cause mortality.	[35]

RDW as a marker of disease severity and clinical manifestation of CAD				
Lippi et al. (2009)	Prospective	2,304 adult patients who were admitted to the emergency department for chest pain suggestive of ACS	The combined measurement of cardiac troponin T and RDW increases diagnostic sensitivity to 99% in diagnosing ACS (diagnostic sensitivity of cardiac troponin T alone was 94%).	[36]
Ephrem and Kanei (2012)	Retrospective	503 patients, with UA or NSTEMI	Elevated RDW is independently associated with higher recourse to CABG in patients presenting with UA or NSTEMI.	[37]
Uysal et al. (2012)	Prospective	370 patients with STEMI versus 156 adults with normal coronary angiography as control groups	High RDW level is associated with STEMI in young patients. There is no difference in the RDW level between groups of elderly patients with STEMI versus patients with normal coronary angiography.	[38]
Isik et al. (2012)	Prospective, cross-sectional	193 patients who underwent coronary angiography for SCAD	RDW is associated with the presence, severity, and complexity of CAD, as determined using SYNTAX score.	[39]
Ma et al. (2013)	Prospective, cohort study	677 patients who underwent coronary angiography due to the presence of angina-like chest pain and/or positive treadmill stress test	RDW is associated with the presence and severity of CAD, as determined using Gensini score.	[40]
Ephrem (2013)	Retrospective	503 patients with UA or NSTEMI	Increased RDW level is independently associated with hospital readmission in patients with UA or NSTEMI.	[41]
Duran et al. (2013)	Prospective, cross-sectional	226 patients with ACS	Elevated RDW level is associated with the absence of coronary collateral vessel (graded according to the Rentrop scoring system) in patients with ACS.	[42]
Akin et al. (2013)	Prospective	580 patients with MI	RDW is associated with severity of CAD (assessed by SYNTAX score) in patients with MI.	[43]
Tanboga et al. (2014)	Retrospective	662 patients with STEMI who underwent primary PCI	RDW level is a predictor of angiographic coronary thrombus burden.	[44]
Akilli et al. (2014)	Prospective	917 patients who underwent dobutamine stress echocardiography	High RDW level is associated with the positive result of dobutamine stress echocardiography and correlates with the extent of ischemia. Moreover, RDW increases the diagnostic accuracy of dobutamine stress echocardiography.	[45]
Bekler et al. (2014)	Retrospective	251 patients with NSTEMI or UA	RDW level is associated with the presence of fragmented QRS complexes in patients with NSTEMI or UA.	[46]
Tanboga et al. (2014)	Prospective, cross-sectional	322 patients with NSTEMI	RDW is a predictor of an impaired coronary collateral circulation (Rentrop grades 0-1) in patients with NSTEMI.	[47]
Acet et al. (2014)	Retrospective	379 patients with STEMI	RDW is an independent predictor of GRACE risk score in patients presented with STEMI.	[48]
Polat et al. (2014)	Retrospective	193 patients with NSTEMI or UA	RDW is a predictor of high GRACE score and in-hospital mortality in patients with NSTEMI or UA.	[49]
Wang et al. (2015)	Retrospective	424 patients with STEMI who underwent primary PCI	High RDW is an independent predictor of the presence of three-branch and left main lesions and thrombotic burden in patients with STEMI.	[50]
Şahin et al. (2015)	Retrospective	326 patients with SCAD who underwent coronary angiography	RDW is independently associated with poor coronary collateral circulation (Rentrop grades 0-1) in patients with SCAD.	[51]
Baysal et al. (2015)	Retrospective	102 patients with STEMI who underwent thrombolysis	RDW level at admission is an independent predictor of the thrombolysis failure in patients with STEMI.	[52]
Li et al. (2015)	Retrospective	203 patients who underwent coronary angiography and dynamic electrocardiography (Holter)	RDW value is independently associated with diurnal corrected QT variation in patients with SCAD.	[53]
Li et al. (2015)	Retrospective	392 patients with CAD	High RDW is associated with elevated FRS in patients with CAD.	[54]
Sahin et al. (2015)	Prospective, cross-sectional	335 patients with NSTEMI	RDW is a predictor of high SYNTAX score but is not associated with long-term mortality in patients with NSTEMI.	[55]

RDW as a predictor of complications and patients outcomes after invasive treatment of CAD				
Karabulut et al. (2012)	Retrospective	556 patients with STEMI treated with primary coronary intervention	RDW is an independent predictor of abnormal reperfusion (TIMI flow less than 3).	[56]
Isik et al. (2012)	Prospective	100 patients with STEMI treated with primary coronary intervention	High RDW is associated with the presence of electrocardiographic no-reflow.	[57]
Akyel et al. (2013)	Retrospective	90 patients with coronary artery grafts who underwent coronary angiography	RDW is a predictor of saphenous vein graft disease.	[58]
Ertaş et al. (2013)	Retrospective	132 patients without history of AF who underwent nonemergency CABG	Preoperative RDW level is a predictor of new-onset AF after surgery.	[59]
Fatemi et al. (2013)	Retrospective	6,689 patients who underwent coronary artery stent implantation	RDW is independently associated with postprocedural major bleeding in patients who underwent PCI with stent implantation.	[60]
Yildiz et al. (2014)	Retrospective	269 patients with SCAD or UA who underwent BMS implantation	Preprocedural RDW is an independent risk factor for ISR in patients who underwent BMS implantation.	[61]
Kurtul et al. (2015)	Prospective	662 patients with ACS treated with PCI	RDW is an independent risk factor for the development of CI-AKI.	[62]
Kurtul et al. (2015)	Retrospective	251 patients with history of BMS implantation who underwent control coronary angiography	RDW level before control coronary angiography is a predictor of the presence of ISR.	[63]
Zhao et al. (2015)	Retrospective	293 patients with SCAD who underwent DES implantation	RDW level at both admission and follow-up is an independent predictor of ISR.	[64]
Akin et al. (2015)	Prospective, cross-sectional	630 patients with STEMI who underwent primary PCI	Authors confirmed that RDW is an independent risk factor for the development of CI-AKI.	[65]
Mizuno et al. (2015)	Prospective	102 patients with STEMI who underwent primary PCI	RDW has potential predictive ability, only if used with Mehran risk score, for CI-AKI in patients with STEMI.	[66]

RDW as a predictor of cardiovascular events in a general population				
Patel et al. (2010)	Meta-analysis	11,827 older adults	In population of older adults RDW is strongly associated with CVD mortality.	[3]
Veeranna et al. (2013)	Prospective, cross-sectional	8,513 adults free of CVD	RDW is independently associated with CAD mortality in a cohort with no preexisting CVD.	[67]
Borné et al. (2014)	Prospective, cohort study	26,820 subjects (aged 45–73) without history of MI or stroke	RDW is associated with increased incidence of fatal ACS, but not with incidence of nonfatal ACS.	[68]
Skjelbakken et al. (2014)	Prospective, cohort study	25,612 participants recruited from a general population	RDW is associated with first-ever MI in general population independent of traditional cardiovascular risk factors and anemia.	[69]

RDW: red blood cell distribution width; CAD: coronary artery disease; HF: heart failure; MI: myocardial infarction; PCI: percutaneous coronary intervention; ACS: acute coronary syndrome; NSTEMI: non-ST elevation myocardial infarction; STEMI: ST elevation myocardial infarction; UA: unstable angina; TIMI: thrombolysis in myocardial infarction; SCAD: stable coronary artery disease; CABG: coronary artery by-pass graft; MACEs: major adverse cardiac events; DES: drug-eluting stent; GRACE: Global Registry of Acute Coronary Events; AF: atrial fibrillation; BMS: bare-metal stent; ISR: in-stent restenosis; FRS: Framingham Risk Score; CVD: cardiovascular disease; CI-AKI: contrast-induced acute kidney injury.
